# Online paediatric chronic pain management: assessing the needs of UK adolescents and parents, using a cross-sectional survey

**DOI:** 10.1177/2049463720940341

**Published:** 2020-07-21

**Authors:** Anna Hurley-Wallace, Daniel E Schoth, Suzanne Lilley, Glyn Williams, Christina Liossi

**Affiliations:** 1 Pain Research Laboratory, School of Psychology, University of Southampton, Southampton, UK; 2 Great Ormond Street Hospital for Children NHS Foundation Trust, London, UK

**Keywords:** Chronic pain, paediatric pain, adolescent health, persistent pain, online intervention, health psychology

## Abstract

**Background:**

Adolescent chronic pain is prevalent, and interdisciplinary treatment is recommended. Although it is well known that technology is a key part of adolescents’ daily lives, there have not been any online, interdisciplinary interventions developed for adolescents with chronic pain in a UK healthcare context. Little is known about how adolescents currently use online resources to manage chronic pain, or what guidance they seek.

**Methods:**

Ninety-five participants from the community answered this mixed-methods, online survey (adolescent n = 54, parent n = 41), which assessed the needs of UK-based adolescents for a new online chronic pain management resource.

**Results:**

Findings indicated that, at the time of the survey, adolescents frequently used social media platforms, such as Instagram, for chronic pain management. Desired techniques for a new interdisciplinary resource for adolescents included ‘advice on explaining chronic pain to others’ (86.7% of adolescents) and sleep hygiene (82.2% of adolescents), though access to a range of pain management techniques was desired. Qualitative results indicated endorsement of a new programme by adolescents and parents.

**Conclusions:**

Adolescents and parents had a positive outlook towards the development of a UK-specific online resource to help manage chronic pain. Such an intervention should aim to be made accessible via the National Health Service. Adolescent use of social media platforms to seek support for chronic pain requires further exploration in future research.

Paediatric chronic pain is an internationally recognised problem; recent estimates indicate 13.2% to 33.8% of adolescents experience multi-site chronic pain, including 16%–19% of UK-based adolescents.^1^ Paediatric chronic pain is often complex and can considerably impair a young persons’ physical, social, emotional and school functioning.^2,3^ Mental health comorbidities, including anxiety and mood disorders, are prevalent and can hinder recovery in children and adolescents with chronic pain.^4–8^ An interdisciplinary approach to paediatric chronic pain management is recommended,^9,10^ and evidence shows that interdisciplinary treatments can improve functional outcomes.^11,12^ However, many families do not have access or cannot travel long distances to clinics.^13^ Self-management using online, remotely delivered, interventions can reduce the number of clinic visits.

A review of psychological interventions to child and adolescent chronic pain showed remotely delivered cognitive–behavioural therapy (CBT) is described positively by patients, with some evidence for reduced pain severity post-treatment for headache but not for mixed chronic pain.^14^ Another review of the availability of e-health tools for paediatric pain identified 53 tools,^15^ 12 of which were intended for chronic pain management. Online adolescent chronic pain programmes successfully developed in the United States and Canada include WebMAP^16^ and iCanCope,™ respectively.^17,18^ There has not, however, been an interdisciplinary multi-modal intervention developed for adolescent chronic pain in a UK context.

Insights from adolescents in the United Kingdom are important as their needs may differ based on their experiences of healthcare, along with their experiences of chronic pain in various social contexts.^19^ For example, in the UK the National Health Service (NHS) offers free access to chronic pain management programmes following GP referral, whereas in the United States insurance companies review requests for specialist consultation.^20^ At a population level, adolescents may identify a range of different priorities and problems which require different solutions to successfully implement an intervention in the real world.^21^ Understanding the needs of this population and gathering their views as potential users of a new resource reflects the Medical Research Council (MRC) guidance for developing complex interventions and integrates the Person-Based Approach.^22^ It is also intuitive to consider parents as stakeholders in development under these frameworks.

While it is well-recognised that adolescents are native internet users,^23^ and social media platforms are a critical part of their daily lives,^24^ little is known about online resources that adolescents use to manage chronic pain, as well as comorbid mental health issues.^4^ Understanding adolescents’ current use of online resources for these purposes is another important part of the context in which adolescents with chronic pain will potentially use a new resource.^21^ Research investigating healthy adolescents’ use of online resources for acute pain management identified that adolescents experienced anxiety around their use, including pain-related anxiety and a mistrust of content.^25^ The use of online resources for pain management has not been investigated in adolescents with chronic pain.

Considering adolescent use of social media for chronic pain management, a scoping review of support-seeking on YouTube found 18 videos targeting adolescents with chronic pain.^26^ Most content covered multidisciplinary and alternative treatments, consistent with interdisciplinary approaches. The videos had 936 comments, and the main message was ‘you are not alone’. These comments indicate many adolescents with chronic pain go online for peer support and also reflect reports that 12 to 15 year olds turn first to YouTube for content that is important to them.^27^

Exactly what guidance adolescents with chronic pain seek online remains unclear. There is also little indication of which online resources are being used except YouTube. Adolescent usage and preferences must be explored to create a viable real-world solution.^21^ This study conducted a needs assessment for a UK-based online, interdisciplinary intervention for managing adolescent chronic pain. The study aimed to investigate (1) which online resources adolescents currently use to manage chronic pain and mental health, (2) which online resources parents use to help them understand their child’s chronic pain, (3) which interdisciplinary techniques adolescents with chronic pain consider most helpful, (4) what content and features adolescents and parents would like to see in a new online chronic pain management intervention, and (5) if reporting high online resource use predicts overall positive outlook, versus negative outlook, towards a new intervention.

## Methods

### Design

The study was an online cross-sectional survey using Qualtrics^®^, including a mixture of closed- and open-ended questions.

### Participants

This was a UK-wide survey of adolescents aged 16 to 18 years with chronic pain and parents of adolescents aged 12 to 18 years. The survey was not distributed directly to 12 to 15 year olds, as this would have required additional consent from parents. While not impossible to attain, the research team decided that a dual consenting process would overcomplicate this study for participants and negatively impact recruitment. Hence, to avoid complication and maintain anonymity, the survey pathways were separated into 16 to 18 year olds self-reporting and parents reporting for the 12 to 18 years age range. A power calculation was conducted, producing a target sample size of 385 (Supplemental Material 1).

For adolescents, inclusion criteria were (1) aged 16 to 18 years and (2) currently experiencing pain of any aetiology which has lasted ⩾3 months^28^ and exclusion criteria: (1) aged ⩽15 years or ⩾19 years, (2) pain lasting less than 3 months of total duration and (3) chronic pain had not been formally diagnosed by a healthcare professional.

For parents, inclusion criteria were (1) parents/guardians of adolescents aged 12 to 18 years, (2) adolescent pain of any aetiology that has lasted ⩾3 months^28^ and exclusion criteria: (1) parents of children aged ⩽11 years or ⩾19 years and (2) adolescent pain has lasted less than 3 months.

### Recruitment

The survey was accessible via an open survey link from 30 May 2019 to 14 October 2019 and advertised UK-wide using posters, social media (Twitter, Facebook, and LinkedIn), relevant charities, patient (or parent) organisations, online forums and ‘letters’ to 93 local newspapers.

Initial screening questions were used to ensure that only adolescents or parents who indicated they met the inclusion criteria could proceed with the survey. A first-stage screening question (on the consent form) ensured all participants were ⩾16 years old; this question also served as a branch to the adolescent or parent version of the survey. A second stage of screening was used to clarify that the young people in question had a chronic pain condition with a duration of ⩾3 months. Qualtrics validation ensured that participants who did not select a valid criterion could not continue the survey and were politely asked to exit.

### Survey and procedure

**Figure 1.**
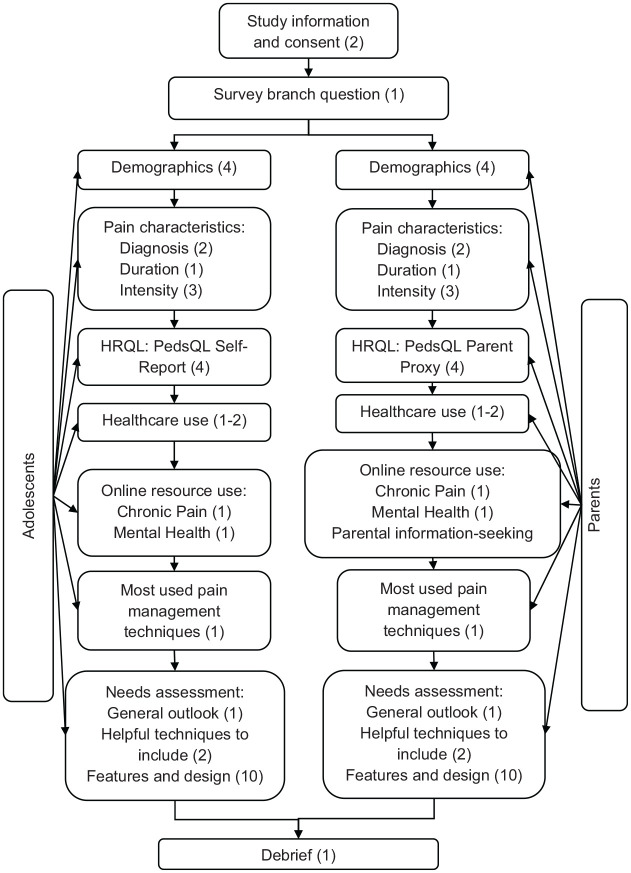
Survey flow for the current study. The diagram shows the survey blocks in flow order, with the number of questions per section indicated in parentheses.

There were 78 questions split between two branches: adolescent and parent versions. Questions in the two branches mirrored each other. The survey took approximately 30 minutes to complete. Participants could return to previous questions and could save the survey and return to complete it within 7 days. If no activity was registered for 7 days, the response was recorded as partially completed. The survey flow is represented in Figure 1.

Full questionnaires details are provided in Supplemental Material 2. Pain diagnoses were collected using the categories outlined for the International Classification of Diseases, 11th Revision (ICD-11),^28^ and intensity was assessed using items from the Brief Pain Inventory (BPI).^29^ Pain duration was also collected. Health-related quality of life (HRQL) was assessed using the PedsQL™ 4.0.^30,31^ Current resource use and needs assessment questions were developed specifically for this study.

### Planned analysis

Statistical analyses were conducted using SPSS version 26. Multiple responses analyses were performed to descriptively summarise participants’ use of online resources for chronic pain and mental health, as well as for preferred content and features (n, %). Pearson chi-square tests were used to explore differences between adolescents and parents in reported resource use and preferred content. Where between-group differences were significant, pairwise comparisons were adjusted using a Bonferroni correction. Note that chi-square tests performed on multiple response data are exploratory as opposed to confirmatory.^32^

For most helpful pain management techniques, participants were asked to rank their top three out of a selection of 19. Responses left empty were considered tied for last place. Missing values were allocated a score of 11.5 in SPSS (mean score of the remaining available ranks (4 + 5 + 6 . . . + 19/16) A rank score was calculated to ascertain the top ranked pain management techniques for adolescents and parent respondents, separately. Lower scores indicate higher ranking.

Independent t-tests were used to compare feature and design preferences between adolescents and parents for scale variables. Categorical responses were compared using Pearson chi-square or Fisher’s Exact Test where >20% of cell counts were <5.

Because 74 out of 78 participants who answered the qualitative question (95%) were positive towards the development of an online intervention, planned logistic regressions to identify predictors of preference became obsolete.

### Qualitative exploration

To explore initial ideas and opinions that adolescents and parents had about a UK-based online chronic pain management programme, a content analysis was conducted on the first question in the needs assessment: ‘what are your initial thoughts about creating a new online resource that could help young people/ you manage chronic pain?’. Responses were first exported to NVivo 12 and cross-tabulated with demographic data, regarding whether the participant was an adolescent or parent, their sex and age.^33^ The content analysis used an inductive approach, in which sentences were the units of analysis.^34^ Open coding followed by categorisation into generic categories and sub-categories was conducted by A.H. (PhD student researching paediatric chronic pain). Categories are labelled with content-characteristic words.^34,35^

## Results

### Participant demographics

A total of 95 UK-based participants, including 54 adolescents and 41 parents, completed this survey.

One-hundred and forty-five individuals accessed the survey, of which 112 completed it. Sixty-one adolescents and 48 parents provided their geographical location. The majority of these were valid UK postcode districts (81.2%) covering multiple regions (England, Wales, Scotland; see Supplemental Material 3. UK Distribution Map). Participants that entered a numeric area code, which appeared to be from outside the United Kingdom, were excluded from analyses (n = 17). Participants that did not enter any location data were included. These participants met screening criteria for chronic pain, and any contributions remained potentially useful. There were eight matched postcodes by district, four of which were cross-matches between the parent and adolescent groups. These matches may or may not have been adolescent–parent dyads. As this was unknown, no additional measures were taken to account for this in data analyses.

Participant demographic and pain characteristic information from the UK sample is displayed in Table 1. Most adolescents were aged 17 (n = 20) or 18 (n = 21) years. Participating parents and guardians were most commonly in the 36 to 55 years age category (95.1%). Most adolescents identified as girls (94.4%). There were three boys, and one person did not identify with any gender category. All of the parents in this sample were women.

**Table 1.** Demographic and pain characteristics for adolescent and parent participants.

	Adolescents (n = 54)	Parents (n = 41)
Age: 16 years, n (%)	13 (24.1)	–
Age: 17 years, n (%)	20 (37.0)	–
Age: 18 years, n (%)	21 (38.9)	–
Age: 18 to 35 years, n (%)	–	1 (2.4)
Age: 36 to 55 years, n (%)	–	39 (95.1)
Age: >55 years, n (%)	–	1 (2.4)
Birth sex, n (%)		
Male	3 (5.6)	0 (0)
Female	51 (94.4)	41 (100)
Gender, n (%)		
Male	2 (3.7)	0 (0)
Female	51 (94.4)	41 (100.0)
Transgender	0 (0)	0 (0)
Does not identify as a male, female, or transgender	1 (1.9)	0 (0)
Chronic pain type^a^ (adolescent), n (%)		
Primary pain	31 (57.4)	21 (51.2)
Cancer pain	0 (0)	1 (2.4)
Post-surgical pain (PSP)	1 (1.9)	1 (2.4)
Neuropathic	8 (14.8)	4 (9.8)
Headache/orofacial	19 (35.2)	9 (22.0)
Visceral	7 (13.0)	5 (12.2)
Musculoskeletal (MSK)	42 (77.8)	38 (92.7)
Pain duration (adolescent), n (%)		
⩾3 months	2 (3.7)	1 (2.4)
⩾6 months	0 (0)	1 (2.4)
⩾1 year	15 (27.8)	6 (14.6)
⩾3 years	12 (22.2)	13 (31.7)
⩾5 years	25 (46.3)	20 (48.8)
Pain intensity–BPI (adolescent), M (SD)	Adolescents (n = 51)	Parent-proxy (n = 41)
Worst in last 24 hours	7.02 (1.33)	6.59 (1.69)
Least in last 24 hours	3.84 (1.77)	4.24 (2.46)
On average	5.59 (1.37)	5.51 (1.33)
Current healthcare use (attending an NHS pain management service), n (%)	Adolescents (n = 50)	Parent report (n = 40)
Yes	11 (22.0)	9 (22.5)
No	39 (78.0)	31 (77.5)
HRQL – PedsQL™ (0–100), (M, SD)	Adolescents (n = 48)	Parent-proxy (n = 38)
Psychosocial summary	33.82 (14.57)	36.62 (14.70)
Emotional scale	31.98 (17.19)	38.03 (19.33)
Social scale	42.29 (20.50)	38.46 (20.17)
School scale	27.19 (17.01)	33.21 (18.33)
Physical summary	23.24 (13.47)	26.07 (17.30)
Total score	30.14 (12.85)	32.95 (14.44)

BPI: Brief Pain Inventory; SD: standard deviation; HRQL: health-related quality of life.

a Participants could select multiple categories for chronic pain type; percentages indicate percent of individual cases that selected the option.

Index of Multiple Deprivation (IMD) scores indicated that the sample were of varied socioeconomic status. The IMD ranks every neighbourhood in England from 1 (most deprived area) to 32,844 (least deprived area). Neighbourhoods in Wales are ranked from 1 to 1909 and Scotland from 1 to 6976. Eighty participants in this sample were from England (IMD; M = 16521), two were from Wales (IMD; M = 967), and seven from Scotland (IMD; M = 4796). IMD rank scores for this sample ranged from 1388 out of 32,844 (10% most deprived in England) to 32,472 out of 32,844 (10% least deprived in England).^36^

The most frequent pain type reported by adolescents and parents was musculoskeletal (MSK) pain (77.8% and 92.7%, respectively). One parent selected cancer pain. The majority of adolescents had been experiencing chronic pain for longer than a year, according to adolescent self-reports (96.3%) and parent-proxy reports (95.2%). The most commonly selected pain duration for both respondent groups was 5 years or longer (adolescents = 46.3%, parents = 48.7%). The HRQL total score for this sample of adolescents with chronic pain (self-reported M = 30.14, SD = 12.85) was low compared to other recent studies of adolescents with chronic pain^37^ (self-reported M = 58.71, SD = 21.58), *t*(90) = –7.79, *p* < .001; very low compared to a healthy 15-year-old sample^38^ (self-reported M = 84.70, SD = 12.70), *t*(335) = –27.52, *p* < .001.

### Use of online resources

Descriptive information about frequency of various resources used to manage chronic pain and mental health is summarised in Tables 2 and 3. Many participants selected multiple online resources for both chronic pain and mental health management. The most frequently selected response by adolescents was that they did not use any websites or apps for pain management (50.0%). The most frequently selected resource by adolescents for managing chronic pain was Instagram (n = 20), although this was not reflected in the parent responses for adolescent Instagram use (n = 5). The majority of parent participants (74.3%) indicated their child did not use any websites or apps for pain management. Exploratory comparison between adolescents and parents did not reveal a significant difference in multiple response entries for chronic pain resources, χ^2^(8) = 15.30, *p* = .054.

**Table 2.** Frequency of adolescent use of online resources and social media platforms for chronic pain management, according to adolescent and parent reports.

Chronic pain resources	Adolescents (n = 48), n (%)	Parents (n = 35), n (%)
Does not use websites/apps	24 (50.0)	26 (74.3)
Instagram	20 (41.7)	5 (14.3)
YouTube	13 (27.1)	6 (17.1)
Facebook	8 (16.7)	4 (11.4)
Online forum	5 (10.4)	2 (5.7)
Uses a different website/app	4 (8.3)	2 (5.7)
Twitter	4 (8.3)	3 (8.6)
Reddit	1 (2.1)	0 (0)
MeeTwo	0 (0)	0 (0)
PainBytes	0 (0)	0 (0)

Participants could select multiple resources; percentages indicate percent of individual cases that selected the option. Resources are listed in descending frequency of selection by adolescents.

**Table 3.** Frequency of adolescent use of online resources and social media platforms for mental health management, according to adolescent and parent reports.

Mental health resources	Adolescents (n = 46), n (%)	Parents (n = 35), n (%)
Does not use websites/apps	23 (50.0)	22 (62.9)
Instagram	16 (34.8)	2 (5.7)
Headspace	10 (21.7)	6 (17.1)
YouTube	9 (19.6)	4 (11.4)
Calm	6 (13.0)	3 (8.6)
Online forum	5 (10.9)	1 (2.9)
Facebook	4 (8.7)	1 (2.9)
Young Minds	2 (4.3)	1 (2.9)
Uses a different website/app	1 (2.2)	2 (5.7)
Twitter	1 (2.2)	1 (2.9)
Reddit	0 (0)	0 (0)
MeeTwo	0 (0)	0 (0)

Participants could select multiple resources; percentages indicate percent of individual cases that selected the option. Resources are listed in descending frequency of selection by adolescents.

For mental health management, the most frequent response from adolescents and parents was that the adolescent did not use any websites or apps for mental health management (50.0% and 62.9%, respectively). The top three most selected resources for mental health management by adolescents were Instagram (n = 16), Headspace (n = 10) and YouTube (n = 9). These selections were not mirrored by the selections made by parents regarding their children’s usage. Adolescent and parent multiple response entries for mental health resources, however, were not significantly different upon statistical exploration, χ^2^(10) = 16.58, *p* = .084.

**Figure 2.**
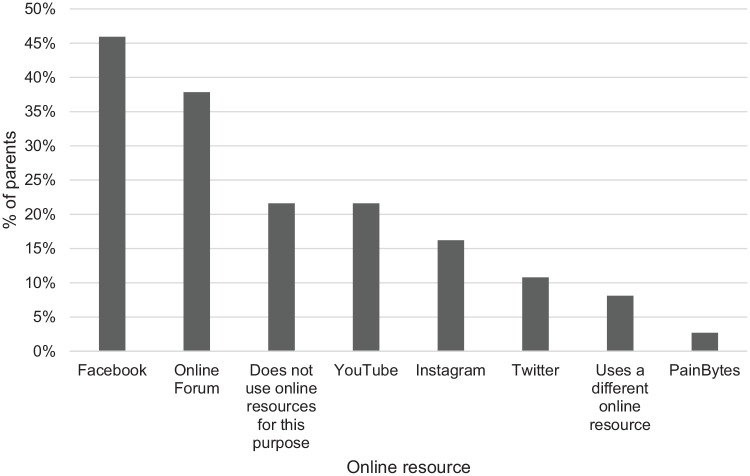
Parent use of online resources to help them understand or manage their adolescents’ chronic pain.

The survey also investigated parent use of online resources to aid their understanding of their child’s chronic pain. As shown in Figure 2, 45.9% of the parents who responded to this question used Facebook as an information resource. Seconding this was use of online forums (37.8%). In the alternative response box, two parents advised that they have used Ehlers-Danlos websites (https://www.ehlers-danlos.org/) as an information resource, and one parent indicated they used the NHS website (https://www.nhs.uk/).

### Most helpful pain management techniques

The top three highest ranked chronic pain management techniques for adolescent respondents (n = 49), were pacing (M rank = 5.9, SD = 4.7), medication (M rank = 6.3, SD = 5.0), and rest (M rank = 6.9, SD = 4.8). Hypnosis and mindfulness were tied for last place within the adolescent group (M rank = 11.5), indicating that none of the adolescents ranked these techniques in their top three. The top three highest ranked chronic pain management techniques by parents (n = 36) were pacing (M rank = 5.9, SD = 4.6), rest (M rank = 7.2, SD = 4.9), and physiotherapy (M rank = 7.5, SD = 4.9). None of the parents ranked biofeedback or exposure therapy in their top three (M rank = 11.5).

### Preferences for content and features in a new programme

Descriptive information regarding the chronic pain management techniques that adolescents and parents indicated they believed would be helpful to include in a new online resource is summarised in Table 4. Many participants selected multiple pain management techniques, although ‘advice on pacing daily activities’ was the most frequently selected by parents (86.1%), followed by ‘methods to improve sleep (80.6%). The most frequently selected option by adolescents was ‘advice on explaining chronic pain to others (e.g. friends and family)’ (86.7%), followed by ‘methods to improve sleep’ (82.2%). Exploratory comparison between adolescent and parent multiple responses revealed a significant between-group difference in preferences for content, χ^2^(19) = 33.49, *p* = .021. Pairwise comparisons using a Bonferroni correction indicated significant differences (*p* *<* .003) for hypnosis and guided imagery, where both options were more frequently selected by parents.

**Table 4.** Most helpful techniques to include in the content of a new online resource for managing chronic pain in adolescents, according to adolescents and parents.

Pain management technique	Adolescents (n = 45), n (%)	Parents (n = 36), n (%)
Advice on explaining chronic pain to others	39 (86.7)	26 (72.2)
Methods to improve sleep	37 (82.2)	29 (80.6)
Advice on pacing daily activities	34 (75.6)	31 (86.1)
Advice on transitioning from ‘paediatric’ to adult healthcare	34 (75.6)	25 (69.4)
Guidance on pain medications	33 (73.3)	19 (52.8)
Pain education	32 (71.1)	20 (55.6)
Advice on pacing for exercise/sports	30 (69.2)	22 (61.1)
Support for returning to school	30 (66.7)	22 (61.1)
Physiotherapy examples	28 (62.2)	18 (50.0)
Examples of other physical pain management techniques	27 (60.0)	23 (63.9)
Massage techniques	22 (48.9)	15 (41.7)
Relaxation and breathing	21 (46.7)	19 (52.8)
Challenging negative thoughts	20 (44.4)	26 (61.9)
Mindfulness/meditation	19 (42.2)	21 (58.3)
Biofeedback	19 (42.2)	12 (33.3)
Exposure therapy	16 (35.6)	12 (33.3)
Art therapy	10 (22.2)	15 (41.7)
Guided imagery/visualisation	5 (11.1)	12 (33.3)
Hypnosis	3 (6.7)	9 (25.0)

Participants could select multiple options; percentages indicate percent of individual cases that selected the option. Items are listed in descending frequency of selection by adolescents.

Other techniques mentioned by adolescents in the optional text entry box included music therapy (n = 2), connecting with others with chronic pain (n = 2) and help with everyday tasks (n = 2). Parents mentioned occupational therapy (n = 1), other CAM techniques (n = 2), and the potential for an online peer support platform for adolescents (n = 2).

### Functional features and design

Regarding programme structure, the majority of adolescent and parent respondents selected they would prefer a ‘flexible structure’, where they could choose which sections they wanted to use (86.7% and 77.1%, respectively). However, when examining between-group differences for all of the available choices for structure (see Supplemental Material 2), there was a statistically significant difference between which choices adolescents and parents selected, two-tailed Fisher Exact *p* *=* .030. The option that differed between respondent groups was ‘I do not mind how the resource is structured’, where 2% of adolescents selected this option compared to 20% of parents. Regarding preference for having professional support while using the intervention (1 = definitely yes to 5 = definitely not), for telephone support, there was a significant difference in preference between adolescent and parent participants, *t*(78) = 2.07, *p* = .042, where adolescents preferred to have telephone support (M = 3.56, SD = 1.08) comparatively to parents (M = 3.06, SD = 1.06). For online professional support, there were no significant differences in preference between adolescents and parents, *t*(78) = –1.31, *p* = .195 (M = 2.09, SD = 1.00 and M = 2.37, SD = .91, respectively). With regard to whether a theme would be appealing or not (response options = ‘yes’, ‘maybe’, or ‘no’), the most common adolescent response was ‘maybe’ (48.9%), and similarly ‘maybe’ was the most common response from parent respondents (40.0%). There was no significant difference between adolescent and parent responses; χ^2^(2) = 2.08, *p* = .403.

The importance of linking an online pain management programme to a hospital or clinic (1 = extremely important to 5 = not at all important) was indicated by adolescent participants to be ‘moderately’ important (M = 3.04, SD = 1.19). There was no significant difference between adolescent and parent responses to the hospital link question, *t*(78) = .90, *p* = .371. The majority of adolescent responses to the question of whether they would prefer video demonstrations of techniques to include a healthcare professional, or a ‘teenage’ patient (there was also an option for no preference), indicated that they would prefer a patient (42.2%). Parent respondents also indicated that they would prefer a patient in video examples (65.7%); no significant between-group differences were indicated; χ^2^(2) = 4.67, *p* = .106. For whether people in video examples should be ‘male, ‘female’ or ‘no preference’, ‘no preference’ was most frequently selected (adolescents = 80.0%, parents = 94.3%), and none of the respondents in either group selected ‘male’. Adolescent and parent responses were not significantly different, Fisher’s Exact *p* *=* .101. Regarding what the ethnicity of the person/people displayed in any video examples should be, the majority of respondents selected no preference (adolescents = 93.3%, parents = 85.7%), where the only other response that was selected was mixed/multiple ethnic groups (adolescents = 6.7%, parents = 14.3%). ‘White’, ‘Asian’ and ‘Black/African/Caribbean’ were not selected by any respondents, and there was no significant difference between respondent groups, two-tailed Fisher Exact *p* = .288.

### Barriers and facilitators to using a new programme

Considering facilitators, two adolescents commented they would like an online programme to include reminders, and barriers mentioned included levels of pain and fatigue, as well as the programme having too much text, or taking too long to work through. The parent comments emphasised to make sure the programme was not patronising or condescending, which was also echoed in comments from two adolescent participants. One parent commented that a barrier to adolescent use might be monitoring, either by the hospital, school or parents.

### Qualitative content analysis

Seventy-eight respondents (adolescents, n = 45; parents, n = 33) answered the initial needs assessment question, ‘what are your initial thoughts about creating a new online resource that could help young people/ you manage chronic pain?’ The adolescent group that answered this question included 1 male and 44 females, and the parent group included 33 females only. The majority of the adolescent group were aged 17 years (42.2%) and the majority of parents were aged between 36 and 55 years (93.9%).

Four generic categories were identified within the data, where the main overarching category can be considered as ‘opinions about a new online resource for young people with chronic pain’, derivative of the research question itself. Categories and sub-categories were condensed from 91 codes identified from the qualitative dataset of responses from both adolescent and parent participants.

An exploratory subgroups analysis was conducted using the generic categories to compare responses from adolescents and parents. All four categories remained clear within parent and adolescent groups. The category that responses were most frequently classified under was ‘good idea’, with 17 responses from adolescents grouped under this category and 21 responses from parents. Adolescents commented more frequently on age-specificity compared to parents (n = 13 and n = 4, respectively).

#### Category 1: good idea

Participant responses were most frequently classified to this category (n = 38), representing the opinion that an online programme for managing chronic pain in adolescents was generally a ‘good’, ‘great’ or ‘excellent’ idea and that participants would be interested in such a programme.

A56: ‘I think a new online resource that could help young people with chronic pain is a brilliant idea’. (Adolescent, 17 years, female)

Two respondents touched on the notion that it would be a good idea to link to NHS services; however, there were not enough comments made about this for ‘NHS linking’ to be considered a sub-category alone.

There was also an element of excitement throughout these comments, indicated by use of superlatives (e.g. ‘amazing’, ‘fantastic’). A few of the adolescents used the word ‘cool’ to indicate excitement.

#### Category 2: helpful

Thirty-five responses were classified under ‘helpful’. This included synonyms of helpful; the other key word used was ‘useful’. An example is quoted in the following. Some comments eluded that adolescents would try anything, rather than showing enthusiasm specifically towards a new resource (see A41). Overall, the comments were positive.

A45: ‘I think it’d be very useful as finding out how to deal with chronic pain is very difficult’. (Adolescent, 18 years, female)

A41: ‘Anything to help even a few people’. (Adolescent, 17 years, female).

##### Sub-category: improving accessibility

This sub-category gave a sense that an online programme would be helpful because it would create a way for adolescents to access help independently. The majority of these comments were from parents.

A23: ‘I think it would help a lot of young people get the help they deserve’. (Parent, 36 to 55 years, female)

A104: ‘. . . Ease of access from home. Not reliant on GP referral etc. – self ownership/ management’. (Parent, 36 to 55 years, female)

##### Sub-category: increasing others’ understanding

A few of the participants’ initial comments revealed a preference for something within a new resource that could help other people understand the chronic pain experience. This is exemplified in the following quote.

A10: ‘Could be useful about helping those without chronic pain to understand’. (Adolescent, 18 years, female)

#### Category 3: adolescent-specific

The need for an age-specific resource for adolescents came through strongly. This category was exemplified well by one of the adolescent participants.

A33: ‘It would be fantastic as there are very little resources for people my age in my area’. (Adolescent, 17 years, female)

##### Sub-category: non-patronising

Within the adolescent-specific category, a few comments were made about ensuring a new programme is not patronising. One participant highlighted whether an intervention is patronising or not depends on the group it is targeting.

A64: ‘It could be good but only if it is targeted appropriately e.g. not patronising’. (Adolescent, 16 years, female)

A42: ‘It can come across offensive because people with chronic pain have tried a lot’. (Adolescent, 17 years, female)

##### Sub-category: connectedness

Under connectedness, there were comments about the need for something to help adolescents feel less alone and about generally connecting with other adolescents who are going through a similar experience. This could be labelled as peer support; however, there was a clear emphasis on knowing people are there empathetically, rather than seeking advice. There were additionally a couple of comments made on social media integration as a way of establishing connections (see example quote A46).

A81: ‘. . . a good idea so that they can compare and make friends with others who understand’. (Parent, 36 to 55 years, female)

A46: ‘It would be beneficial; using social media platforms would be good for that’. (Adolescent, 16 years, female)

#### Category 4: concerns

While there were few concerns or negative comments made (n = 12), it is important that negative comments be acknowledged in light of developing an online intervention. Some respondents made comments that were too vague to interpret exactly what the concern was.

A30: ‘It’s a good idea as long as it’s good, well-meaning and doesn’t do harm’. (Adolescent, 18 years, female)

These types of comments could not be categorised under a specific sub-header. Many of these responses were juxtaposed, such as the comment by participant A30. Outside of more general comments, an underlying concern was the relevance of intervention content.

##### Sub-category: content relevance

Concerns about the relevance of the content in an online resource for adolescent chronic pain management were evident. These included comments about the broad range of chronic pain conditions and that different people manage differently. Participants also commented on tangible support over self-management.

A101: ‘Not sure if really helpful – [a] lot of resources, no idea of reality – need practical help and a life’. (Parent, 36–55 years, female)

A98: ‘Would need to be wide-ranging to cover different causes of pain; could make it unwieldy to use’. (Parent, 36–55 years, female)

## Discussion

The aim of this study was to conduct a UK-wide needs assessment for an online, interdisciplinary intervention for paediatric chronic pain management, the results of which offer valuable insight into the needs of adolescents regarding online chronic pain management. Even though the survey was conducted in the UK, the results can inform aspects of the development of online interventions in other Western countries.

Considering online resources used to manage chronic pain and mental health issues, the majority of adolescents and parents indicated adolescents did not use online resources for either purpose. This is surprising given positive evaluations of mindfulness-based apps such as Headspace.^39–41^ Only 10 adolescents indicated they used Headspace, and one indicated ‘Calm’ (another commercially available app). Social media resources were selected much more frequently by adolescents than parents, possibly because parents are generationally less familiar with social media and do not necessarily know the resources their children use.^23^ While psychological factors play a key role in the maintenance of paediatric chronic pain,^9,42^ there seems to be low endorsement of available psychology-based tools to manage concurrent mental health issues.

Prior research reveals adolescents often access YouTube for important information and specifically for chronic pain information.^26,27^ The present results support this as 27% of adolescents indicated they use YouTube as a support resource. However, this study highlighted Instagram as another important resource for chronic pain, selected by 42% of adolescents. While Instagram originated as a platform for uploading still photographs, the latest versions (2020) allow uploads of video content (up to 1 minute) and for direct messages between users. Additional video content can be uploaded by business users to Instagram TV. Mirroring the previous investigation of YouTube content,^26^ Instagram content on adolescent chronic pain warrants further exploration. It is concerning that the current lack of a trusted online resource for adolescent chronic pain management may lead to adolescents accessing content that is not evidence-based or accurate, which could perpetuate problems. Recent media reports note insufficient monitoring of harmful, self-injury promoting social media content, despite efforts to eradicate it.^43^ A solution may be the creation of an evidence-based resource for adolescent chronic pain that can be made accessible via the NHS or a linked service.

Considering parent use of online resources to help them understand their child’s chronic pain, findings indicated 46% use Facebook as a support resource. This is another area of interest concerning whether information shared on Facebook groups is evidence- based. The second most used resource by parents was online forums. This supports previous investigations of parental online communication on forums for paediatric Complex Regional Pain Syndrome (CRPS) for informational and empathetic support.^44^ It is possible that parents in the present study of mixed chronic pain used forums for similar reasons. Only one parent used the NHS website as an information resource, which may indicate an increased need for empathetic support over informational.

Interdisciplinary pain management techniques (not online) ranked as the most helpful differed somewhat between adolescents and parents. Medication was ranked as the second most helpful intervention by adolescents but was not highly ranked by parents. This may indicate medication use in older adolescents is high, despite a lack of evidence that pharmacological interventions are effective as a standalone treatment for chronic pain.^45–49^ Pacing was the top ranked technique by both groups, and rest was also ranked in the top three for both groups. The majority of this sample were not attending a specialist pain clinic at the time of the survey, which may explain why medication and rest were ranked high, while psychological treatments were ranked low. Psychological techniques are less likely to be cited by healthcare professionals working outside of specialist chronic pain services.^50^ However, data on whether participants attended a specialist clinic in the past were not collected.

Regarding preferred chronic pain management techniques adolescents and their parents wanted to see in a new programme, many adolescents selected ‘advice on explaining chronic pain to others’ (87%). This may be because adolescents with chronic pain often struggle with social functioning^3,51,52^ and are at increased risk of peer victimisation compared to healthy peers.^3^ While it would be useful to include social advice in a new online programme, this finding may reflect a need for community and school-based interventions that target peer understanding.

Most participants indicated they wanted access to ‘methods to improve sleep’ (82% of adolescents and 80% of parents), reflecting prior research findings that 54% of adolescents with chronic pain report insomnia symptoms.^53^ In relation to online interventions, currently available CBT-based chronic pain management has not been found to significantly improve sleep outcomes in adolescents.^54^ Researchers from this study suggested that, as reductions in pain and disability were not associated with improved sleep, poor sleep is likely fuelled by a variety of factors. Content on improving sleep requires more focus in new programmes; examples of sleep hygiene techniques for adolescents are available in the wider literature.^55^

Concerning preferred interdisciplinary techniques for a new resource, the majority of participants selected several techniques out of the 19 available to select. Clearly, access to a range of techniques is desired, though it is debateable how many techniques can feasibly be included in one online resource. This need for choice of techniques is similar to the concept of a ‘pain toolbox’, which is successfully utilised in CBT-based online interventions for adolescent chronic pain.^16^

While qualitative responses lacked depth, four clear categories were identified. The first two categories (‘good idea’ and ‘helpful’) were expressed strongly by both adolescent and parent respondents, indicating an overall positive outlook towards online modalities of pain management. New, evidence-based, targeted resources for chronic pain self-management are likely to be welcomed by adolescents and parents.

One pertinent response from the adolescent qualitative data was that there is nothing age-specific available. There is a clear need for resources aimed at adolescents, which is not patronising, and allows them to connect in a similar way to social media. Social media is a critical part of adolescent’s lives and different platforms are used for different purposes.^24^ The current study indicates adolescents are seeking a platform that is specific to chronic pain. Recent reports of YouTube use in 12 to 15 year olds note that 52% use vloggers as a source of online content and inspiration.^23^ An important part of chronic pain management for some adolescents may be through following others with painful conditions. Interactive, peer support platforms have been successfully developed for paediatric chronic pain and arthritis (iPeer2Peer)^56,57^ as standalone programmes. A pilot study of iPeer2Peer, including 28 adolescents with chronic pain, found those who completed the series of 10 Skype-based calls with a peer mentor significantly improved their coping abilities and self-management skills.^56^ There is potential for elements of peer support to be integrated within interdisciplinary programmes, which may help adolescents to feel more connected and supported in their self-management.

This need for an adolescent-specific resource may also highlight a lack of acknowledgement that adolescents and children have different needs. Previous research on health information-seeking found that adolescents with pain seek information online as a way of assuming independence over their health.^25^ Promisingly, the focus of online interventions that have been developed for adolescents with chronic pain in the United States and Canada has been on self-management.^16–18^ The overarching message is that adolescence represents a unique stage of physical, social and emotional development,^58–60^ and interventions should be targeted appropriately.

Several study limitations should be noted. First, the target sample size was not met, and therefore quantitative, descriptive results are unlikely to be generalizable to the wider population of UK-based adolescents with chronic pain, and only representative of respondents.^61^ There were no implications of sample size for the qualitative content analysis. The content analysis answered the intended research question regarding adolescent and parent opinions towards a new online resource, thereby meeting the informational needs of the study.^62^ Second, while it is expected an adolescent chronic pain sample would contain more girls than boys based on prevalence statistics,^63^ 94% of the adolescent sample were girls. Data regarding the sex of the adolescents that parents were responding about were not collected. As such, these findings should not be generalised to adolescent boys.

## Conclusion

The results of the current study indicate that use of online resources and social media for managing chronic pain is common in adolescents, with many turning to Instagram and YouTube for content and support. Overall, development of a new online resource for chronic pain was endorsed by adolescents and parents, with a need for connectedness and age-specific content emphasised. Access to a range of interdisciplinary techniques is desired. New online interventions for adolescents in the UK should aim to be accessible via the NHS as an evidence-based resource. Novel research exploring how adolescents use social media platforms to manage chronic pain and seek support is recommended.

## Supplemental Material

Supplementary_Material_1._Sample_size_calculation – Supplemental material for Online paediatric chronic pain management: assessing the needs of UK adolescents and parents, using a cross-sectional survey Click here for additional data file. Supplemental material, Supplementary_Material_1._Sample_size_calculation for Online paediatric chronic pain management: assessing the needs of UK adolescents and parents, using a cross-sectional survey by Anna Hurley-Wallace, Daniel E Schoth, Suzanne Lilley, Glyn Williams and Christina Liossi in British Journal of Pain

Supplementary_Material_2._Details_of_questionnaires_administered – Supplemental material for Online paediatric chronic pain management: assessing the needs of UK adolescents and parents, using a cross-sectional survey Click here for additional data file. Supplemental material, Supplementary_Material_2._Details_of_questionnaires_administered for Online paediatric chronic pain management: assessing the needs of UK adolescents and parents, using a cross-sectional survey by Anna Hurley-Wallace, Daniel E Schoth, Suzanne Lilley, Glyn Williams and Christina Liossi in British Journal of Pain

Supplementary_Material_3._UK_Distribution_Map – Supplemental material for Online paediatric chronic pain management: assessing the needs of UK adolescents and parents, using a cross-sectional survey Click here for additional data file. Supplemental material, Supplementary_Material_3._UK_Distribution_Map for Online paediatric chronic pain management: assessing the needs of UK adolescents and parents, using a cross-sectional survey by Anna Hurley-Wallace, Daniel E Schoth, Suzanne Lilley, Glyn Williams and Christina Liossi in British Journal of Pain
