# Supplementary material for: Online paediatric chronic pain management: assessing the needs of UK adolescents and parents, using a cross-sectional survey
Source: Br J Pain. 2020 Jul 21;15(3):312–25. doi: 10.1177/2049463720940341 (PMC8339947; doi:10.1177/2049463720940341)
Supplement: Supplementary_Material_1._Sample_size_calculation – Supplemental material for Online paediatric chronic pain management: assessing the needs of UK adolescents and parents, using a cross-sectional survey [file Supplementary_Material_1._Sample_size_calculation.docx]

# Sample size calculation

A target sample size was calculated to estimate how many participants would be required to produce results that accurately represent the UK-based adolescents with CP. Primary aims of the study were to find out which online resources adolescents and parents currently use to manage chronic pain and mental health issues, and what content and features adolescents and parents would like to see in a new online intervention. As these were categorical selections, a sample size calculation for categorical data was used:

$$n= \frac{p\left( 1-p \right)z^{2}}{e^{2}}$$

n = required sample size, p = population variance, *e* = percentage maximum error, *z* = value corresponding to level of confidence

With a confidence level set at 95% (*z* *=* 1.96), margin of error (*e*) set at 5%, and variance (p) maximised at .50, a representative sample size was calculated as 385 complete responses in total (1, 2).

This calculation assumes parent responses are representative of adolescents aged 12 to 15 years, and those aged 16 to 18 years would answer the survey themselves. Mathematical correction was not needed, as the required sample size of 385 does not exceed 5% of the UK population of adolescents with CP (3-5).

References

1. Qualtrics. Sample Size Calculator 2019 [Available from: <https://www.qualtrics.com/blog/calculating-sample-size/>.

2. Taherdoost H. Determining Sample Size; How to Calculate Survey Sample Size. 2017.

3. Gobina I, Villberg J, Välimaa R, Tynjälä J, Whitehead R, Cosma A, et al. Prevalence of self-reported chronic pain among adolescents: Evidence from 42 countries and regions. European Journal of Pain. 2019;23(2):316-26.

4. Cochran WG. Sampling techniques. 3rd ed. New York: John Wiley & Sons; 1977.

5. Kotrlik J, Higgins C. Organizational research: Determining appropriate sample size in survey research appropriate sample size in survey research. Information technology, learning, and performance journal. 2001;19(1):43.
