# Supplementary material for: Online paediatric chronic pain management: assessing the needs of UK adolescents and parents, using a cross-sectional survey
Source: Br J Pain. 2020 Jul 21;15(3):312–25. doi: 10.1177/2049463720940341 (PMC8339947; doi:10.1177/2049463720940341)
Supplement: Supplementary_Material_2._Details_of_questionnaires_administered – Supplemental material for Online paediatric chronic pain management: assessing the needs of UK adolescents and parents, using a cross-sectional survey [file Supplementary_Material_2._Details_of_questionnaires_administered.docx]

# Details of questionnaires administered in this survey

## Pain characteristics

Pain condition was classified as per ICD-11 diagnostic categories (1) i.e., chronic primary pain, chronic cancer pain, chronic postsurgical and posttraumatic pain, chronic neuropathic pain, chronic headache and orofacial pain, chronic visceral pain, and chronic musculoskeletal pain. Multiple selections were allowed as some patients’ chronic pain overlaps two or more categories, where one of the categories will be defined as the ‘primary parent’ diagnosis.

Participants were asked who diagnosed their chronic pain (options: GP, Consultant, Nurse, or ‘Someone else’). If they selected ‘Someone else’ they were asked to specify whom, using a text input box.

The next question related to pain duration. Duration could be selected from ≥ 3-months, ≥ 6-months, ≥ 1-year, ≥ 3-years, or ≥ 5-years. There were also options to select < 3-months or < 4-weeks. However, participants that selected these options were politely advised to exit the survey, as a pain duration of < 3-months does not match the criteria for chronic pain outlined for the ICD-11 (1).

Pain intensity was then assessed using the numerical rating scales from the Brief Pain Inventory (BPI) (2) which ask patients to rate their pain at its worst in the last 24 hours, at its least in the last 24 hours, and ‘on average’ (0 = no pain to 10 = pain as bad as you can imagine). The BPI, although was initially developed to assess cancer related pain it has been validated, and used widely, in non-malignant CP samples (3, 4). An acceptable level of internal consistency was achieved for adolescent BPI scores (α = .784), and parent-proxy scores (α = .793).

## Health-related quality of life

Health-related quality of life (HRQL) was measured using the Pediatric Quality of Life Inventory (PedsQL™ 4.0) (5, 6), which is a validated scale that measures HRQL in children and young people in different age bands, as developmentally appropriate. The Child Self-Report is available for ages 5 to 7, 8 to 12, and 13 to 18 years, and the Parent Proxy-Report is available for ages 2 to 4, 5 to 7, 8 to 12, and 13 to 18 years. The version used in the current study targeted adolescents aged 13 to 18 years, and the parent proxy-report was used for the parent branch of the survey. The adolescent PedsQL™ contains 23 items and four subscales (physical, emotional, social and school functioning). HRQL total scores were computed as per guidance from the PedsQL™ 4.0. A psychosocial summary score can be computed using the emotional, social and school functioning subscales, and the physical summary is comprised of scores from the physical subscale only. Transformed HRQL scores range from zero to 100, with a higher score indicating better HRQL. The PedsQL™ is widely used to assess HRQL in healthy (7-9) and clinical populations of children and adolescents; for example in ADHD (10-12) and chronic fatigue (13, 14), as well as specifically in paediatric chronic pain research (15-20). Adolescent and parent proxy reports from the present sample had high internal consistency (α = .895 and α = .908, respectively).

## Healthcare use

Adolescents and parents were asked if they/ their child currently (at the time of taking the survey) attended an ‘NHS-based pain management service or program’ (‘Yes’ or ‘No’). Participants that selected ‘Yes’ were additionally asked ‘which healthcare professionals have been helping you to manage chronic pain?’ They could select multiple options for this, which included: GP, consultant, nurse, occupational therapist, physiotherapist, and psychologist. Participants that answered ‘No’ to the first question were re-directed to the subsequent section.

## Online resource use

The next section asked about which resources adolescents used to manage chronic pain and mental health. Adolescents and parents could select from a range of options or input something different. There were 10 different resources available to select for chronic pain, and 12 resources available to select in the mental health management; these included a variety of apps and websites, as well as social media platforms. For both chronic pain management, and mental health management, options were presented in a randomised order. An adolescent volunteer (female, 15 years old) was asked for her input about the resources available to select for mental health management before they were finalised.

## Parental information-seeking

Parents were additionally asked ‘As a parent/ guardian, do you use any online resources to help you understand or manage your teenager’s chronic pain?’ Nine options were available to select, including the option to state: ‘I do not use online resources to understand/ manage my teenager’s chronic pain’ or ‘I use a different online resource’. The other options included apps, websites, and social media resources. Options were presented in a randomised order and multiple options could be selected.

## Most used pain management techniques

Following this, adolescents and parents were asked to rank their/ their child’s top three most useful pain management techniques in general (as opposed to online management only). There were 19 techniques available from which participants were asked to rank their top three (1 = most helpful, 2 = 2^nd^ most helpful, 3 = 3^rd^ most helpful). A range of pain management techniques from medicine, nursing, occupational therapy, psychology, and complimentary alternative medicine (CAM) were included in this selection. The following options were presented in a randomised order:

| Medication/ pain-killers | Getting good night’s sleep |
| --- | --- |
| Getting help and support with school work | Improving my understanding of chronic pain |
| Improving other people’s understanding of chronic pain | Physiotherapy exercises |
| Keeping active | Pacing myself |
| Relaxation and breathing | Mindfulness and/ or meditation |
| Other physical pain management methods (e.g. using TENS, thermal analgesia, desensitisation) | Psychological therapy - Cognitive Behavioural Therapy (CBT) |
| Hypnosis | Massage |
| Biofeedback (increasing awareness and modifying physiological processes e.g. heart rate) | Psychological therapy - Exposure therapy |
| Art therapy | Guided imagery and/or visualization |
| Rest |  |

## Needs assessment

This section begun with the question ‘What are your initial thoughts about creating a new online resource that could help young people/ you manage chronic pain?’ which was a text input response question (qualitative). The main section then comprised of a series of questions about preferred techniques to be included in online CP management (resource content), followed by questions about features and design.

Participants first selected which techniques they believed would be helpful to include in an online pain management resource for teenagers from a randomised selection of 19 techniques. These included techniques from multiple disciplines, for which multiple options could be selected from the following list:

| Advice/ guidance on pain medications | Methods to improve sleep |
| --- | --- |
| Support for returning to school | Advice on explaining chronic pain to others (e.g. friends and family) |
| An explanation of what chronic pain is ('pain education') | Physiotherapy examples |
| Advice on how to pace yourself in daily activities | Advice on how to pace yourself for exercise/ sport |
| Relaxation and breathing techniques | Mindfulness and/ or meditation techniques |
| Advice on transitioning from 'paediatric' (child) to adult healthcare | Examples of other physical pain management methods (e.g. using TENS, thermal analgesia, desensitisation) |
| Challenging and restructuring negative thoughts | Hypnosis |
| Massage techniques | Biofeedback (increasing awareness and modifying physiological processes e.g. heart rate) |
| Exposure therapy techniques (i.e. gradually exposing yourself to situations that you would usually avoid) | Art therapy |
| Guided imagery and/or visualization |  |

Participants were then asked if there was any technique or therapy (not yet mentioned) that would be especially useful to have access to at home (text input response). Further questions addressed resource structure and design. Participants were asked what sort of structure they would like, and could select from (i) a flexible structure (can chose what sections they want to use), (ii) set structure (to be completed over a number of days/ weeks), (iii) ‘I do not mind how the resource is structured’, (iv) ‘I would like something else’ (this contained a text input field). They were then asked if they would like professional adjunctive support whilst they/ their teenager was accessing the intervention. Questions were asked separately for telephone and online support, and scored as follows: 1 = definitely yes, 2 = probably yes, 3 = might or might not, 4 = probably not, 5 = definitely not. They were then asked if it would be appealing for the program to have a theme (for example, a travel theme), where response options were ‘yes’, ‘maybe’ and ‘no’. Subsequent questions asked how important the design of an online resource directed at teenagers is, whether it was important that the new program was associated with a hospital or clinic, and whether pictures and videos are important for online pain management in teenagers (1 = extremely important, 2 = very important, 3 = moderately important, 4 = slightly important, 5 = not at all important). Participants were also asked about who should feature in video content (a healthcare professional, a (teenage) patient, or ‘no preference’), and whether the person in videos should be of a particular gender (male, female or ‘no preference’) or ethnicity (White, Mixed/ multiple ethnicities, Asian/ Asian British, Black/ African/ Caribbean/ Black British, or ‘no preference’). Comments on facilitators and barriers to using an online resource to manage chronic pain were collected in the penultimate question, which asked ‘Is there anything that could motivate or prevent you/ your teenager from using an online pain programme on a regular basis?’, with options to answer ‘not that I know of’ or ‘yes (please state)’, which contained a text response box to specify. The final question was also a text entry question, which asked for any additional comments about online chronic pain management for young people.

References

1. Treede RD, Rief W, Barke A, Aziz Q, Bennett MI, Benoliel R, et al. A classification of chronic pain for ICD-11:. PAIN. 2015:1.

2. Cleeland CS, Ryan K. The brief pain inventory. Pain Research Group. 1991:143-7.

3. Tan G, Jensen MP, Thornby JI, Shanti BF. Validation of the brief pain inventory for chronic nonmalignant pain. The Journal of Pain. 2004;5(2):133-7.

4. Keller S, Bann CM, Dodd SL, Schein J, Mendoza TR, Cleeland CS. Validity of the Brief Pain Inventory for Use in Documenting the Outcomes of Patients With Noncancer Pain. The Clinical Journal of Pain. 2004;20(5):309-18.

5. Varni JW, Burwinkle TM, Seid M, Skarr D. The PedsQL™* 4.0 as a Pediatric Population Health Measure: Feasibility, Reliability, and Validity. Ambulatory Pediatrics. 2003;3(6):329-41.

6. Varni JW, Seid M, Kurtin PS. PedsQL™ 4.0: Reliability and Validity of the Pediatric Quality of Life Inventory™ Version 4.0 Generic Core Scales in Healthy and Patient Populations. Medical Care. 2001;39(8):800-12.

7. Motamed-Gorji N, Qorbani M, Nikkho F, Asadi M, Motlagh ME, Safari O, et al. Association of screen time and physical activity with health-related quality of life in Iranian children and adolescents. Health and Quality of Life Outcomes. 2019;17(1):2.

8. Bazzano AN, Anderson CE, Hylton C, Gustat J. Effect of mindfulness and yoga on quality of life for elementary school students and teachers: Results of a randomized controlled school-based study. Psychology Research and Behavior Management. 2018;11.

9. Lam KC, Valier ARS, Bay RC, McLeod TCV. A unique patient population? Health-related quality of life in adolescent athletes versus general, healthy adolescent individuals. J Athl Train. 2013;48(2):233-41.

10. Al-Habib D, Alhaidar F, Alzayed I, Youssef R. Consistency of child self-reports with parent proxy reports on the quality of life of children with attention-deficit/hyperactivity disorder in Riyadh, 2016. Journal of Family and Community Medicine. 2019;26(1):9-16.

11. Erbilgin Gün S, Kilincaslan A. Quality of Life Among Children and Adolescents With Tourette Disorder and Comorbid ADHD: A Clinical Controlled Study. Journal of Attention Disorders. 2018;23(8):817-27.

12. Yürümez E, Kiliç B. Relationship Between Sleep Problems and Quality of Life in Children With ADHD. Journal of attention disorders. 2013;20.

13. Roma M, Marden CL, Flaherty MAK, Jasion SE, Cranston EM, Rowe PC. Impaired Health-Related Quality of Life in Adolescent Myalgic Encephalomyelitis/Chronic Fatigue Syndrome: The Impact of Core Symptoms. Frontiers in Pediatrics. 2019;7(26).

14. Winger A, Kvarstein G, Wyller VB, Ekstedt M, Sulheim D, Fagermoen E, et al. Health related quality of life in adolescents with chronic fatigue syndrome: a cross-sectional study. Health and quality of life outcomes. 2015;13:96-.

15. Varni JW, Shulman RJ, Self MM, Nurko S, Saps M, Saeed SA, et al. Symptom Profiles in Patients With Irritable Bowel Syndrome or Functional Abdominal Pain Compared With Healthy Controls. Journal of Pediatric Gastroenterology and Nutrition. 2015;61(3):323-9.

16. Slater SK, Kashikar-Zuck SM, Allen JR, LeCates SL, Kabbouche MA, O’Brien HL, et al. Psychiatric comorbidity in pediatric chronic daily headache. Cephalalgia. 2012;32(15):1116-22.

17. Kalapurakkel S, Carpino EA, Lebel A, Simons LE. "Pain Can't Stop Me": Examining Pain Self-Efficacy and Acceptance as Resilience Processes Among Youth With Chronic Headache. J Pediatr Psychol. 2015;40(9):926-33.

18. Yetwin AK, Mahrer NE, John C, Gold JI. Does Pain Intensity Matter? The Relation between Coping and Quality of Life in Pediatric Patients with Chronic Pain. Journal of Pediatric Nursing. 2018;40:7-13.

19. Tran ST, Jastrowski Mano KE, Hainsworth KR, Medrano GR, Anderson Khan K, Weisman SJ, et al. Distinct Influences of Anxiety and Pain Catastrophizing on Functional Outcomes in Children and Adolescents With Chronic Pain. J Pediatr Psychol. 2015;40(8):744-55.

20. Simons LE, Claar RL, Logan DL. Chronic pain in adolescence: parental responses, adolescent coping, and their impact on adolescent's pain behaviors. J Pediatr Psychol. 2008;33(8):894-904.
