# Supplementary figures and images for: Online paediatric chronic pain management: assessing the needs of UK adolescents and parents, using a cross-sectional survey
Source: Br J Pain. 2020 Jul 21;15(3):312–25. doi: 10.1177/2049463720940341 (PMC8339947; doi:10.1177/2049463720940341)

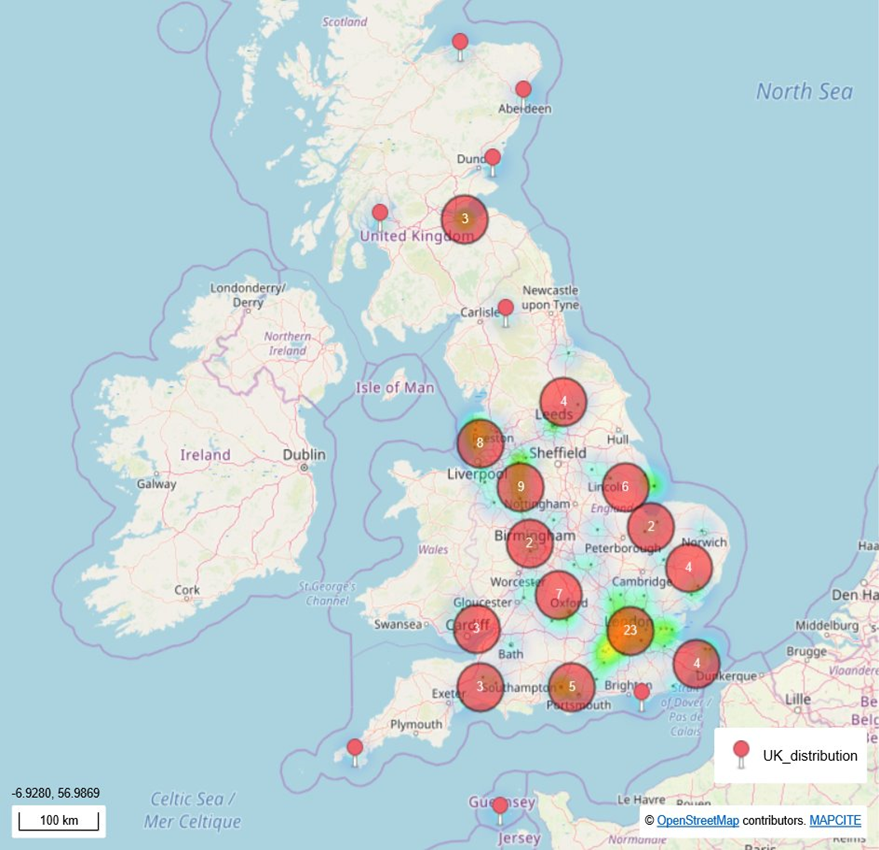

Supplement: Supplementary_Material_3._UK_Distribution_Map – Supplemental material for Online paediatric chronic pain management: assessing the needs of UK adolescents and parents, using a cross-sectional survey [file Supplementary_Material_3._UK_Distribution_Map.tif]
